# Improving lactose digestion and symptoms of lactose intolerance with a novel galacto-oligosaccharide (RP-G28): a randomized, double-blind clinical trial

**DOI:** 10.1186/1475-2891-12-160

**Published:** 2013-12-13

**Authors:** Dennis A Savaiano, Andrew J Ritter, Todd R Klaenhammer, Gareth M James, Amy T Longcore, Justin R Chandler, W Allan Walker, Howard L Foyt

**Affiliations:** 1 Department of Nutrition Science, Purdue University, Stone Hall, Rm 213, 700 W. State Street, West Lafayette, IN 47907-2059, USA; 2 Ritter Pharmaceuticals, Los Angeles, CA, USA; 3 Departments of Food, Bioprocessing and Nutrition Sciences, North Carolina State University, Raleigh, USA; 4 Marshall School of Business, University of Southern California, Los Angeles, USA; 5 Massachusetts General Hospital for Children, Charlestown, MA, USA

**Keywords:** Lactose intolerance, Colonic adaptation, Galacto-oligosaccharides, GOS, Hydrogen breath test, RP-G28, Microbiome, Microflora

## Abstract

**Background:**

Lactose intolerance (LI) is a common medical problem with limited treatment options. The primary symptoms are abdominal pain, diarrhea, bloating, flatulence, and cramping. Limiting dairy foods to reduce symptoms contributes to low calcium intake and the risk for chronic disease. Adaptation of the colon bacteria to effectively metabolize lactose is a novel and potentially useful approach to improve lactose digestion and tolerance. RP-G28 is novel galacto-oligosaccharide (GOS) being investigated to improve lactose digestion and the symptoms of lactose intolerance in affected patients.

**Methods:**

A randomized, double-blind, parallel group, placebo-controlled study was conducted at 2 sites in the United States. RP-G28 or placebo was administered to 85 patients with LI for 35 days. Post-treatment, subjects reintroduced dairy into their daily diets and were followed for 30 additional days to evaluate lactose digestion as measured by hydrogen production and symptom improvements via a patient-reported symptom assessment instrument.

**Results:**

Lactose digestion and symptoms of LI trended toward improvement on RP-G28 at the end of treatment and 30 days post-treatment. A reduction in abdominal pain was also demonstrated in the study results. Fifty percent of RP-G28 subjects with abdominal pain at baseline reported no abdominal pain at the end of treatment and 30 days post treatment (p = 0.0190). RP-G28 subjects were also six times more likely to claim lactose tolerance post-treatment once dairy foods had been re-introduced into their diets (p = 0.0389).

**Conclusions:**

Efficacy trends and favorable safety/tolerability findings suggest that RP-G28 appears to be a potentially useful approach for improving lactose digestion and LI symptoms. The concurrent reduction in abdominal pain and improved overall tolerance could be a meaningful benefit to lactose intolerant individuals.

**Study registration:**

ClinicalTrials.gov NCT01113619.

## Introduction

Lactose intolerance (LI) is a common medical problem that significantly impacts the lives of affected individuals. Patients report symptoms including abdominal pain, diarrhea, bloating, flatulence, and abdominal cramping. The inability to digest lactose, lactose maldigestion (LM), occurs when the concentration of lactase enzyme is reduced in the brush border of the small bowel mucosa. This reduction typically begins early in childhood. Seventy-five percent of the world’s population are maldigesters and, as dairy consumption spreads globally [1], these individuals are susceptible to develop sensitivity to lactose, i.e. lactose intolerance. In the United States, it is estimated that up to 80 million Americans have the potential for lactose intolerance [1]. Symptoms of intolerance result when undigested lactose moves to the colon where it is fermented to produce acetate, carbon dioxide, hydrogen gas and methane. The osmotic effects of lactose and its fermentation products cause the symptoms most frequently associated with LI [2].

The most common advice that physicians give patients with LI is to avoid dairy foods (Objective Insights, June 2012, unpublished data). However, this advice carries a significant nutritional risk. Dairy foods are excellent sources of calcium, potassium, vitamin D, B vitamins and high quality protein. In 2010, the United States Department of Health and Human Services reviewed 55 observational studies from 1967 to 2009 and indicated that low dietary milk and dairy intake was a risk factor for bone fracture, osteoporosis and other adverse health outcomes [3-5]. Dietary calcium supplements did not consistently increase bone mineral density or reduce fracture risk. At the 2010 NIH Consensus Conference on Lactose Intolerance and Health, there was a strong call for additional research that would encourage dairy food consumption while limiting symptoms of intolerance [5]. In addition to health consequences, dairy avoidance decreases the types and the abundance of lactose-digesting bacteria in the digestive tract and makes sufferers even more sensitive when accidentally exposed to dairy products.

Colonic bacterial adaptation in response to prebiotic therapy is one of the most promising new treatments for many gastrointestinal conditions including LI [6]. When microbial adaptation in the human intestinal tract occurs in a patient with LM, the altered population of anaerobic and microaerophilic bacteria increases intraluminal beta-galactosidase activity, thereby enhancing digestion and reducing the production of fermentation products [7]. As early as 1993, adaptation of the colon bacteria by increasing the exposure to lactose was a suggested approach by Briet et al [8] to improve lactose digestion and tolerance. In 1996, Hertzler and Savaiano demonstrated significant improvement in lactose digestion and tolerance and elevation of fecal beta-galactosidase due to colonic adaptation [9].

Galacto-oligosaccharides (GOS) are similar to lactose but contain 2-4 galactose units per molecule. GOS are non-digestible and not absorbed into the blood stream [10]. Colonic adaptation, as a result of GOS administration, has been reported since the 1990s [11-14]; however, no effective treatments for lactose intolerance using this mechanism have been developed. The hypothesis for the present study is that administration of RP-G28, a GOS, will shift colonic bacterial metabolism such that there will be both an improvement in lactose digestion and improved tolerance to an orally-administered lactose load.

## Methods

A first-in-human, proof-of-concept study was conducted with RP-G28 between March 2011 and November 2011. RP-G28 is a proprietary product that is greater than 95% galacto-oligosaccharide. The empirical formal is: C_(n+2)6_H _22+10n_ O _(n+2)5_. The study objectives were to evaluate the effectiveness, safety, and tolerability of RP-G28 in subjects with lactose intolerance. The study was a randomized, double-blind, parallel group, placebo-controlled study conducted at 2 sites in the United States. The trial was approved by the Institutional Review Board (IRB) on February 15, 2011, and the research was carried out in accordance with the clinical research practices defined in the Good Clinical Practice (GCP) guidelines of the International Conference on Harmonisation (ICH).

**Figure 1:**
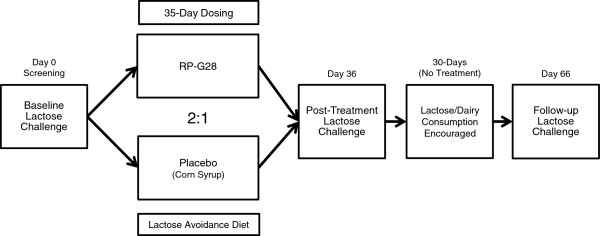
Study design.

An overview of the Study Design is shown in Figure 1. Once informed consent was obtained, eligible subjects underwent screening assessments. Key inclusion criteria included adults ages 18 to 64 with current or recent self-reported history of dairy intolerance of at least 1-month duration. In order to confirm lactose intolerance and study participation, subjects underwent a 25-gram lactose challenge in the clinic. Lactose intolerance symptoms and hydrogen production via hydrogen breath test (HBT) were assessed in the clinic for 6 hours post-lactose challenge. Eligible subjects were required to demonstrate a minimum symptom score and a positive hydrogen breath test in order to be eligible for randomization. A positive HBT was defined as a hydrogen gas elevation of 20 parts per million (ppm) at 2 time-points within the 6 hours following a lactose-loading dose. Key exclusion criteria included diabetes mellitus, disorders known to affect GI motility such as gastroparesis or amyloidosis, disorders with GI symptoms such as irritable bowel syndrome, inflammatory bowel disease and celiac disease, or a history of surgery known to alter the normal function of the GI tract. At Visit 2, subjects meeting all eligibility criteria were randomized 2:1 [RP-G28:placebo] based on the randomization sequence. A 3-digit randomization code, which identified the treatment, was assigned to each subject sequentially based on the order in which the subject qualified. The pharmacist was unblinded to subject randomization; however, the clinical study staff, Medical Monitor, Study Monitor and Sponsor remained blinded throughout the entire study.

The investigational product (IP), RP-G28 or placebo, was provided to subjects as a liquid in high density polyethylene (HDPE) bottles. Each HDPE bottle contained 1 dose of the IP. Subjects added water to the bottle to dilute the IP prior to ingestion. Investigational product was dosed with a meal. Placebo comparator was corn syrup with a similar consistency and sweetness and used the same dosing schedule and packaging as RP-G28. The dose of RP-G28 and Placebo was escalated in 5-day increments according to a fixed schedule from 1.5 grams per day (given once daily) to 15 grams per day (given as 7.5 grams twice daily). The precise schedule of dose escalation can be found in Table 1. During the 35-day treatment period, all subjects were asked to avoid dairy products. After completion of the treatment period, subjects were followed for an additional 30 days and instructed to reintroduce dairy foods back into their diets.

**Table 1:** Schedule of dose escalation for both RP-G28 and Placebo subjects

**Dose escalation schedule**		
**Day**	**Breakfast**	**Dinner**
1–5	-	1.5 g
6–10	-	3.0 g
11–15	-	6.0 g
16–20	1.5 g	6.0 g
21–25	3.0 g	6.0 g
26–30	6.0 g	6.0 g
31–35	7.5 g	7.5 g

To demonstrate effectiveness of RP-G28, subjects underwent two additional 25-gram lactose challenges on Day 36 (immediately post-treatment) and Day 66 (30 days post-treatment). Evaluation of lactose digestion was measured by hydrogen production in the HBT and evaluation of lactose intolerance symptoms was measured by the subject’s self-assessment of symptoms over the 6 hours following the lactose challenge.

The primary efficacy endpoints for the study were change from baseline to Day 36 in 6-hour HBT total hydrogen production and change from baseline (6 hour timepoint) to Day 36 (6 hour timepoint) in lactose intolerance symptom assessment total score during lactose challenge. The primary safety and tolerability endpoints for this study were: adverse events (AEs), vital signs, clinical laboratory results, physical examinations. Table 2 shows both the primary endpoints and secondary endpoints established for the trial. Additional details on each of the efficacy endpoints are noted below:

**Table 2:** Primary and Secondary endpoints

**Primary and Secondary endpoints**	
**Type**	**Description**
Primary	Change from baseline to Day 36 in 6-hour HBT total hydrogen production;
Primary	Change from baseline (6-hour timepoint) to Day 36 (6-hour timepoint) in lactose intolerance symptom assessment total score during lactose challenge.
Primary-Safety	Adverse events (AEs), Vital signs, Clincal laboratory results, physical examinations
Secondary	Change from baseline to Day 66 in 6-hour HBT total hydrogen production
Secondary	Change from baseline (6-hour timepoint) to Day 66 (6-hour timepoint) in lactose intolerance symptom assessment total score during lactose challenge
Secondary	Change from baseline (6-hour timepoint) to Day 36 (6-hour timepoint) in individual lactose intolerance symptom assessment categories (ie, abdominal pain, bloating, flatulence, diarrhea, and abdominal cramping)
Secondary	Change from baseline (6-hour timepoint) to Day 66 (6-hour timepoint) in individual lactose intolerance symptom assessment categories (ie, abdominal pain, bloating, flatulence, diarrhea, and abdominal cramping)
Secondary	Responder analysis, comparing RP-G28 group to placebo group, based on numbers (percent) of subjects achieving a 50% reduction in symptoms (total and individual scores, 6 hours) at Day 36 compared with the baseline lactose challenge and at Day 66 compared with the baseline lactose challenge
Secondary	Patient Global Assessment

### Hydrogen Breath Test (HBT)

Lactose digestion was measured by breath hydrogen production. HBT machines were provided to each site and were properly calibrated to minimize variability in the data. To reduce other external factors on the HBT results, subjects were asked to refrain from using mouthwash or toothpaste and to refrain from strenuous exercise on the evening before and the morning of the clinic visit. Subjects were instructed to fast for at least 8 hours prior to the lactose challenge. On the evening before the HBT, subjects were given dinner restrictions, particularly to have a low intake of sugar, carbohydrate, and fiber as well as to avoid all dairy products. Over the 6-hour assessment period post-lactose challenge, subjects did not smoke, sleep, lie down, or engage in strenuous exercise. At the start of the HBT, subjects exhaled into a gas collection bag, and the breath concentrations of hydrogen, methane, and CO_2_ were measured (baseline, Hour 0). The subject then ingested 25 grams of lactose in a liquid solution and breath samples were collected hourly for 6 hours. Total hydrogen production was calculated as the sum of the hydrogen levels in ppm above baseline at each time-point.

### Lactose intolerance symptom assessment

In parallel to the HBT, symptoms of LI [i.e., abdominal pain, bloating, flatulence, diarrhea, and cramping] were collected at 3-and 6-hour time-points following the 25 gram, in-clinic lactose challenge at baseline, Day 36 (end of treatment) and Day 66 (30 days post-treatment). Severity of symptoms was evaluated using an 11-point Numerical Rating Scale from 0 (none) to 10 (worst).

### Patient Global Assessment (PGA)

The subject’s perception of lactose tolerance was captured using a quality of life questionnaire on Day 66.

### Safety assessments

Adverse Events (AEs) were collected and monitored from the time a subject received the IP until the end of the study or early termination. In general, any clinically significant changes from baseline were considered AEs. Serious Adverse Events (SAEs) were AEs which resulted in death, were life-threatening, required inpatient hospitalization, resulted in a significant incapacity, or were considered to be an important medical event.

### Sample size

The power calculations for each endpoint were performed independently. With the calculation of total hydrogen production, literature reports indicate a lactose challenge of 6, 12, or 20 g result in mean total 8-hour hydrogen productions of 145, 292, and 488 ppm above baseline, respectively, with SDs of approximately 126 ppm [15]. Assuming the mean total baseline value for all subjects was near 488 ppm, and the mean total result after 35 days of treatment was near 145 and 292 ppm for the RP-G28 and placebo groups, respectively, a sample size of 44 subjects in the RP-G28 group and 22 subjects in the placebo group would have provided > 99% power to detect a statistically significant difference with a p-value of 0.05.

For the power calculation of the lactose intolerance symptom assessment, benchmarks were established using a literature reference with a similar study design [16]. Assuming a total mean symptom score baseline of 14.5 and a post-treatment score of 3.7 in the RP-G28 group and 8.1 in the placebo group, a total of 66 subjects randomized 2:1 (treatment to placebo) provided 76% power to detect a difference with a p-value of 0.05. Allowing for a 20% drop out rate, approximately 80 to 100 subjects were to have been enrolled.

A randomization ratio of 2:1 was being used to maximize the exposure to RP-G28 in this Phase 2 study.

### Data analysis

The intent to treat (ITT) population was defined as all randomized subjects who received at least one dose of study medication. All safety analyses were performed using the ITT population. The efficacy analysis was conducted on the per protocol (PP) population because the PP population included all subjects who were randomized and completed through Day 36. Subjects excluded from the PP population did not submit Day 36 data and therefore there was no data to use for efficacy analysis. At any point, if data for a measure were missing, the data remained missing. No imputation of missing data was performed.

Using the PP population (N = 62), the median change from baseline (Day 0) at Day 36 in breath hydrogen levels and the change from baseline (Day 0) in each symptom at Day 36 was calculated. For each symptom, those subjects who reported having had the symptom at Day 0 and reported at least a 50% reduction in severity were classified as “responders.” A Chi square test was performed to analyze the difference in the responder rates between the placebo and RP-G28 groups. Analysis of covariance (ANCOVA) was performed to analyze the change from baseline at Day 36 for median breath hydrogen and for each symptom. For each ANCOVA, treatment, study center, and the treatment/study center interaction were included as factors and the appropriate baseline measurement was included as a covariate.

## Results

**Figure 2:**
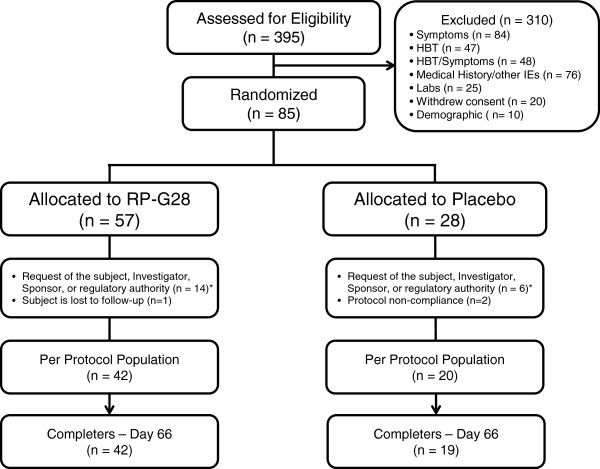
Subject disposition.

There were 395 subjects screened. 310 subjects were excluded, and 85 subjects were randomized. Of those, 57 were randomized to RP-G28 and 28 to placebo. In the randomized population, 42% were male, the mean age of 41 years and the mean BMI of 27.1 kg/m^2^. 38% of the participants were Asian, 26% were African-American, 15% were White, and 21% were other. There were no obvious differences in the two treatment groups. Due to a discoloration of a batch of study medication, 18 subjects (12 in the RP-G28 group and 6 in the placebo group, since the study was double blinded) were prematurely withdrawn by the Sponsor before Day 36. None of these subjects were included in any of the efficacy analyses since they did not complete Day 36. Five additional subjects withdrew from the study between randomization and Day 36 for the following reasons: withdrew consent (n = 2; both in RP-G28 group), lost to follow-up (n = 1; RP-G28 group), and protocol non-compliance (n = 2; placebo group). 62 subjects (42 subjects in the RP-G28 group and 20 subjects in the placebo group) completed through Day 36 of the study and comprised the per protocol population. Between Day 36 and the final visit on Day 66, one additional patient (in the placebo group) was lost to follow-up. See Figure 2 for an overview of subject disposition.

### HBT results

The mean and median breath hydrogen levels post lactose load on Day 0 (baseline) and Day 36 are shown in Table 3. For both treatment groups, the peak hydrogen production occurred 2 hours after lactose challenge on Day 0 and Day 36. Both mean and median hydrogen production decreased to a greater extent after treatment with RP-G28 as compared to the placebo group. While the p value for differences in hydrogen production was not statistically significant due to variation, the likelihood of five consecutive experimental hourly values (hours 2 through 6) falling below the control values is one in 32, a significant difference by simple odds-ratio analysis.

**Table 3:** Differences in Mean and Median Hydrogen Production at Days 0 and 36, p = .1909

		**H2 amount–hourly means**							**Total ∆**
		**0**	**1**	**2**	**3**	**4**	**5**	**6**	
**RP-G28 (ppm)**	Day 0	0	32.17	87.17	81.43	77.17	68.02	63.95	
	Day 36	0	40.90	77.05	75.24	62.79	60.05	48.98	
	∆	0	8.74	-10.12	-6.19	-14.38	-7.98	-14.98	-44.90
**Placebo (ppm)**	Day 0	0	50.05	75.25	85.40	68.35	67.35	52.75	
	Day 36	0	43.10	89.20	82.15	80.85	61.05	51.65	
	∆	0	-6.95	13.95	-3.25	12.50	-6.30	-1.10	8.85
		**H2 amount–hourly medians**							**Total ∆**
		**0**	**1**	**2**	**3**	**4**	**5**	**6**	
**RP-G28 (ppm)**	Day 0	0	18.5	79.5	76.0	67.5	62.0	58.5	
	Day 36	0	15.0	71.0	60.0	56.0	50.5	34.5	
	∆	0	-3.5	-8.5	-16.0	-11.5	-11.5	-24.0	-75.0
**Placebo (ppm)**	Day 0	0	19.5	82.0	76.5	51.5	79.0	57.5	
	Day 36	0	21.0	75.0	76.5	51.5	71.0	54.5	
	∆	0	1.5	-7.0	0.0	0.0	-8.0	-3.0	-16.5

ppm = parts per million, H2 = hydrogen.

### Lactose intolerance symptom assessment results

**Figure 3:**
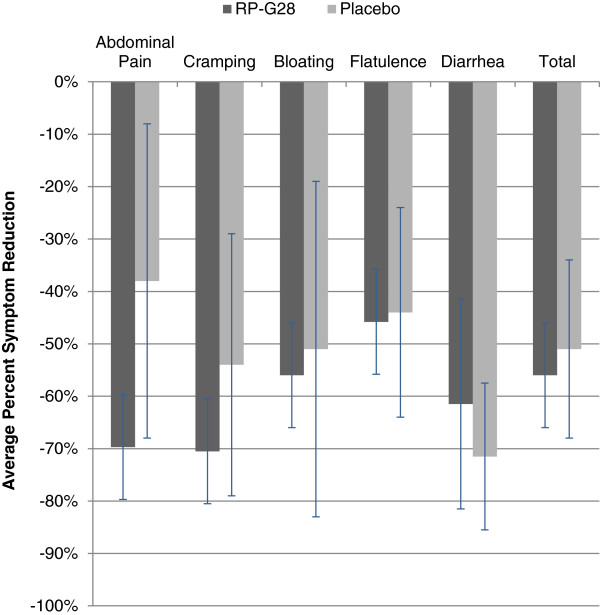
**Average percent change in patient-reported symptoms between Days 0 and 36.** RP-G28 (n = 42). Placebo (n = 20). p = 0.7291

**Figure 4:**
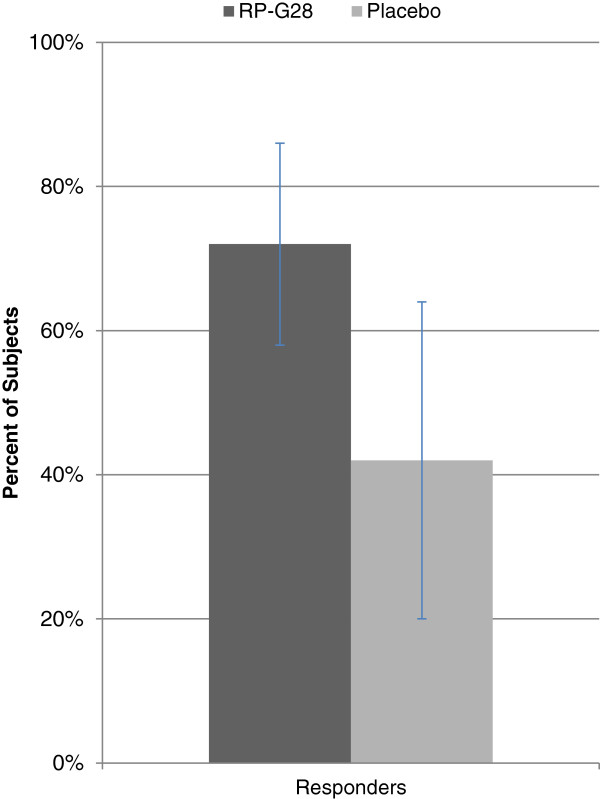
**Subjects reporting >50% decrease in abdominal pain (“Responders”).** RP-G28 (n = 36). Placebo (n = 19). Subjects with no abdominal pain at baseline were excluded from the analysis. p = 0.0288.

Symptoms of abdominal pain, cramping, bloating, and flatulence trended toward improvement with RP-G28 as compared to the placebo (Figure 3). Responder data for abdominal pain (i.e., subjects who at Day 36 reported over a 50% decrease in abdominal pain from baseline), showed that 72% of subjects on RP-G28 responded to treatment compared to 28% on placebo. (p = 0.0288; Figure 4).

**Figure 5:**
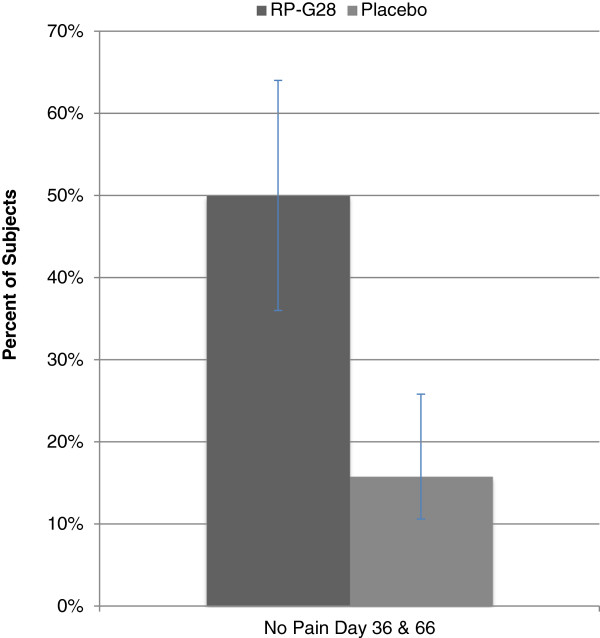
**Subjects reporting no abdominal pain on Day 36 and 66.** RP-G28 (n = 36), Placebo (n = 19), p = 0.0190.

Additionally, 50% of patients on RP-G28 who reported abdominal pain at baseline reported no abdominal pain on both Day 36 and Day 66 (n = 36). In the placebo group, only 17% of subjects experienced no abdominal pain on both Day 36 and Day 66 (n = 19). This result was statistically significant (p = 0.0190; Figure 5).

### Patient Global Assessment results

**Figure 6:**
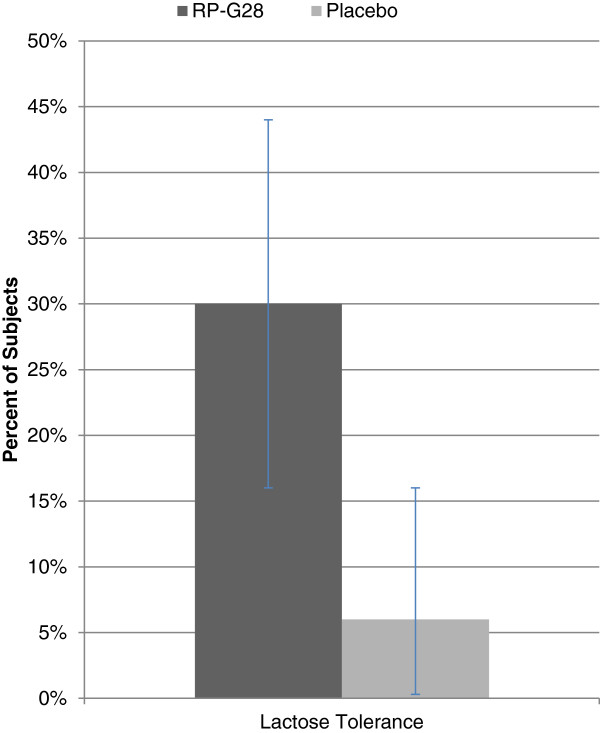
**Patient Global Assessment on Day 66 showing percent of subjects claiming lactose tolerance.** RP-G28 (n = 40). Placebo (n = 18). p = 0.0389.

After completion of study treatment at Day 36, subjects were encouraged to re-introduce dairy foods into their diets. Thirty days later, subjects were questioned whether or not they considered themselves lactose tolerant. 30% of the RP-G28 (n = 40) group considered themselves lactose tolerant as opposed to only 6% (n = 18) of the placebo group (Figure 6). This finding was statistically significant (p = 0.0389). The presence of abdominal pain for the PGA treatment group declined over the course of the trial (Table 4). After treatment, 64% of this group no longer experienced abdominal pain. After 30 days of dairy re-introduction, 82% of this same group no longer experienced abdominal pain.

**Table 4:** Presence of abdominal pain in subjects on RP-G28 reporting lactose tolerance in the patent global assessment

	**Abdominal pain**	**No abdominal pain**	**% with symptoms**
**Baseline**	11	0	100%
**Day 36**	4	7	36%
**Day 66**	2	9	18%

### Safety results

RP-G28 was well tolerated. The most common AEs were headache, dizziness, nausea, upper respiratory tract infection, nasal congestion and pain. All AEs were mild or moderate in severity, and event occurrence was generally distributed over the treatment and post-treatment follow-up phase. Most of the AEs were thought to be unrelated to blinded investigational product by the investigator. There were no Serious Adverse Events (SAEs). No clinically significant changes or findings were noted from clinical laboratory evaluations, vital sign measurements, physical examinations, or 12-lead ECGs.

## Discussion

Current strategies for treatment of lactose intolerance include avoidance of lactose-containing dairy foods (milks, soft cheeses and ice creams) and the use of lactase enzyme supplements with dairy ingestion. By avoiding dairy products, many lactose intolerant individuals in the US and other developed countries have lower bone density [17]. Low calcium consumption increases the risk for chronic diseases, most notably osteoporosis and its sequelae [18]. Colonic adaptation, to allow adequate dairy consumption without uncomfortable clinical symptoms, holds significant promise for lactose intolerant patients [6,7,9,11-13]. The mechanism for this adaptation includes the selection of a higher diversity and concentration of lactose-metabolizing species of bacteria, induction of microbial beta-galactosidase [9] and enhanced utilization of hydrogen gas produced during fermentation [7]. As a result, lactose can be rapidly metabolized by intestinal flora with limited production of uncomfortable clinical symptoms. Adaptation of the gut can turn symptomatic LI patients into lactose digesters [9] despite the underlying genetically-controlled biology of low intestinal lactase activity.

GOS preparations have been shown to stimulate microfloral changes [19]. However, these preparation have high levels of lactose contaminates and are not ideal for lactose intolerant patients. This clinical study is the first to specifically look at the effect of RP-G28, a high quality GOS, on lactose intolerant individuals. Results of the trial show that RP-G28 is effective at improving the clinical symptoms and digestion of lactose while maintaining an excellent safety and tolerability profile.

The variation in breath hydrogen seen over the 6-hours post lactose challenge is typical for a breath hydrogen curve. While the differences are not large, it is clear that the RP-G28 group had lower levels of breath hydrogen from Hours 2 to 6. In the placebo group, these differences are not apparent. Although the HBT is useful to diagnose LM, it is not used in clinical practice for the assessment of LI severity and changes in HBT do not directly correlate to changes in LI symptoms. Thus, HBT would not provide clinically meaningful information to the clinician or patient.

This study further explores methods for identifying meaningful treatment benefits to patients coping with LI. A 0-10 Numerical Rating Scale of symptoms following a lactose challenge was utilized. Mean differences between baseline and follow-up symptom scores were intended to be a straightforward method to evaluate symptom improvement. The challenge with mean scores is that they may show small numerical difference among an individual patient’s symptom reduction even though the improvement may have been a robust and meaningful effect for the patient. Also, in evaluating a total mean symptom score, symptom categories that may be responsive are “diluted” by the unresponsiveness of other categories. As a result, it’s important to evaluate symptoms individually and use additional methods to objectively quantify clinically meaningful results.

There were two results which were signals of RP-G28’s meaningful benefit to patients: the Patient’s Global Assessment and the number of subjects reporting abdominal pain at Days 36 and 66 (Figures 5, 6). The global assessment of a patient’s symptom improvement was used to evaluate patient response to therapy. Patient Global Assessment data are widely accepted to provide the most reproducible clinical responses [20] in evaluating treatment efficacy in patients with functional GI disorders. In this study, RP-G28 treated subjects were six times more likely, versus placebo, to report being lactose tolerant 30 days after discontinuing treatment; this result was statistically significant. This finding provides evidence suggestive of the strength of RP-G28’s effect beyond the acute treatment phase.

In patients reporting abdominal pain at baseline following a 25-gram lactose load, half reported a zero on the pain scale immediately after treatment and 30 days post-treatment. It is reasonable to conclude that the development of tolerance to lactose is associated with a significant reduction in the incidence of abdominal pain in patients with symptomatic lactose intolerance when treated with RP-G28. Literature suggests that functional abdominal pain is the key symptom that drives other gastrointestinal symptoms because it occurs after stimulation of multimodal afferent neural pathways [21]. This appears to be mediated by responses to distension and may be important in the development of symptoms, not just in patients with LI, but in irritable bowel syndrome and inflammatory bowel disease.

Future studies should consider capturing the time-course, intensity, frequency and duration of LI symptoms following lactose ingestion. The benign adverse event safety profile observed in this trial suggests that higher doses of RP-G28 may be well-tolerated and have a more pronounced effect on the improvement of lactose intolerance symptoms experienced by subjects administered an acute treatment of RP-G28.

The significant elimination of abdominal pain post-treatment and 30-days post treatment combined with a benign safety profile support continued clinical evaluation of RP-G28. RP-G28 has potential to allow people the ability to regularly consume dairy foods without experiencing symptoms of LI.

## Competing interests

The authors declare that they have no competing interests.

## Authors’ contribution

All authors participated in the preparation of the manuscript. DAS: Clinical trial design, data analysis. AJR: Clinical trial design, clinical trial oversight, data analysis. TRK: Clinical trial design, data analysis. GMJ: Statistical analysis. ATL: Clinical trial design. JRC: Statistical analysis, data analysis. WAW: Clinical trial design, data analysis. HLF: Clinical trial design, clinical trial oversight, data analysis. All authors read and approved the final manuscript.
